# Crazy-Paving Pattern Due to Herpetic Pneumonia in a Patient with Mycosis Fungoides: A Case Report

**Published:** 2017-06

**Authors:** Ramin Sami, Sara Aeini, Raheleh Sadegh

**Affiliations:** 1 Department of Pulmonology, School of Medicine, Isfahan University of Medical Sciences, Isfahan, Iran.,; 2 Faculty of Medicine, Qazvin University of Medical Sciences, Qazvin, Iran,; 3 Faculty of Medicine, Isfahan University of Medical Sciences, Isfahan, Iran

**Keywords:** Herpes simplex virus 1, Pneumonia, Mycosis Fungoides

## Abstract

Herpetic pneumonia in immune deficient patients could be fatal if not treated. Considering the low prevalence of this disease, computed tomography (CT) scan findings of this condition are not well elucidated. This report describes the CT scan findings of a patient with immune system deficiency due to mycosis fungoides, and pneumonia caused by herpes simplex virus 1 (HSV-1). Bilateral alveolar infiltration with crazy-paving pattern was observed on CT scan of the lungs. The scattered crazy-paving pattern noted in the CT scan of the lungs could be suggestive of herpetic pneumonia in immunocompromised patients presenting with lung infections.

## INTRODUCTION

Respiratory infections are the most common invasive tissue infections in patients with immune deficiency (1). Pneumonia caused by herpes simplex virus type 1 (HSV-1) is uncommon, but has been described as a fatal infection in immunocompromised individuals (1). Since the prevalence of this disease is quite low (except for patients with severe immunodeficiency), its radiographic findings have not been extensively described. However, early diagnosis is vital as the mortality rate associated with this disease, especially in untreated patients, is considerably high.

Mycosis fungoides (MF) is the most common type of non-Hodgkin’s T-cell lymphoma which primarily affects the skin but eventually involves the lymph nodes and other organs (2) leading to humoral and cellular immune deficiency (3). Further, the risk of opportunistic infections, especially herpes, is significantly higher in these patients compared to healthy individuals (4). Pulmonary involvement is noted in 40 % to 60 % of cases of mycosis fungoides (5).

In immunocompromised patients presenting with symptoms of infectious diseases and pulmonary involvement, chest radiography is not sufficient and computed tomography (CT) scan should be suggested. Although, some of the findings of CT scan are not specific, it helps to narrow down the differential diagnosis. However, findings of CT scan in herpetic pneumonia are not widely reported.

In this case study, we report the CT scan findings of herpetic pneumonia in a patient with mycosis fungoides.

## CASE SUMMARIES

The patient was a 60-year-old man, who was diagnosed with MF two years ago, and had been on retinoid compounds since one year. He had been experiencing weakness, lethargy, productive cough, dyspnea, chills, and fever, since two weeks. There were no complaints of headache, vomiting or urinary abnormality. On admission, he had mild respiratory distress and his vital signs were as follows: blood pressure (BP): 110/70 mmHg, pulse rate (PR): 112/min, respiratory rate (RR): 28 breaths/min, oral temperature (OT): 38.8 °C, and oxygen saturation: 84 %.

Multiple lesions suggestive of candidiasis were found on oral examination, while diffuse pulmonary crackles were heard on lung auscultation. Cardiac and extremity examinations revealed only tachycardia and erythroderma/alopecia, respectively. Sputum samples were collected for microbial evaluations. Significant laboratory findings included a C-reactive protein level of 86 mg/L, white blood cells (WBCs): 23600/μL (polymorphonuclear leukocytes: 40 %, lymphocytes: 60 %), Hemoglobin (Hgb): 8.1 gr/dL, and erythrocyte sedimentation rate (ESR): 70 mm/hr.

Chest radiography revealed patchy bilateral alveolar infiltration of the lungs. The patient was hospitalized with a diagnosis of pneumonia, and broad-spectrum antibiotic therapy was initiated. Swab samples of throat were sent for ruling out influenza. Considering the seasonal prevalence, oseltamivir was prescribed.

**Figure 1.**
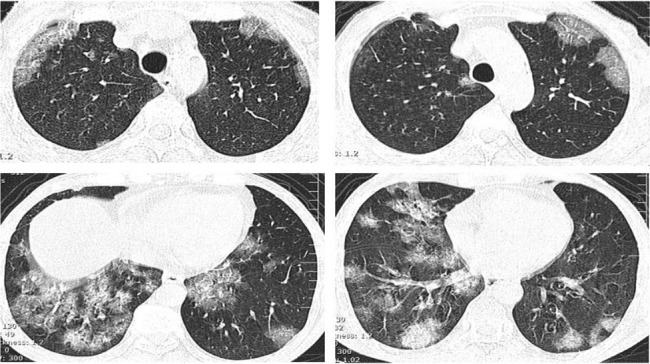
Chest CT shows Crazy-paving pattern and ground glass opacity with lower lobes and peripheral distribution.

A CT scan of the lungs was advised. Thin section CT scan of the lungs revealed diffuse alveolar infiltration and scattered crazy-paving pattern, predominantly in lung bases (Figure 1). After 48 hours, the patient’s condition deteriorated and his blood pressure gradually decreased. Immunological tests to rule out autoimmune diseases were all negative and the level of IgE was normal. Sputum and blood culture were negative. Human immunodeficiency virus (HIV) antibody test was also negative. Bronchoscopy was performed to rule out opportunistic infections and other non-infectious causes; alveolar lavage and lung biopsy were carried out.

On the third day of hospitalization, a gradual reduction in blood pressure was noted and the patient lost his consciousness. Due to respiratory distress and loss of consciousness, the patient was intubated. Real-time polymerase chain reaction (Real-Time PCR) evaluation of the alveolar lavage sample tested positive for HSV-1 (7,000,000 copies/ml), but negative for other herpes viruses and respiratory viruses such as influenza. Smear and lavage culture did not reveal anything significant. Unfortunately, the patient passed away due to respiratory failure on the fifth day of the hospitalization, before antiviral therapy could be initiated. Lung biopsy did not reveal anything significant except for non-specific inflammation.

## DISCUSSION

Opportunistic infections such as mycosis fungoides in immunocompromised patients can significantly influence the treatment outcomes. Early detection and treatment of these infections can thus be life-saving. Respiratory infections are the most common invasive tissue infections in patients with immune deficiency and hence, herpetic pneumonia should be suspected in immunocompromised patients with pulmonary involvement, especially if there are evidences of oral and/or pharyngeal involvement (1). Herpes simplex virus, not only can cause pneumonia in babies but also in immunocompromised adults (1). In herpetic pneumonia, lung CT scan results are non-specific, and varied patterns such as diffuse ground glass opacity, diffuse nodules, and interlobular septal thickening, have been reported (6). In some of studies, ground glass opacity pattern has been reported as the main CT finding of herpetic pneumonia (6, 7). In the study by Chong et al (6), the ground glass opacity was bilateral and diffuse or multi-focal, in all cases of herpetic pneumonia. In our study, bilateral involvement of pulmonary bases was observed.

Ground glass opacity and crazy-paving patterns were the main patterns noted in CT scan in the current study. Crazy-paving pattern refers to the combination of thickening of the interlobular septa and ground glass regions, which occurs due to alveolar involvement. Although this finding was initially observed in patients with pulmonary alveolar proteinosis, it was later noted in many other diseases including infections, pulmonary edema, sarcoidosis, lipoid pneumonia, and diffuse pulmonary hemorrhage (8, 9). Viruses causing this pattern include adenovirus, cytomegalovirus, herpes simplex and influenza (10).

Considering the extensive differential diagnosis in infected immunocompromised patients, invasive methods to detect pathogens are preferred; bronchoscopic study and biopsy should be performed in all such cases. Although herpetic pneumonia has a rapid progression, patients can be saved if bronchoscopy is performed on the first day, and before any treatment. Virus isolation in bronchial lavage samples (besides cytologic and histologic findings) is generally considered to be confirmatory for herpetic pneumonia. However, there are no definitive criteria specified to aid in the diagnosis of this disease. Some of the researchers suggest that identification of the virus in the bronchial lavage fluid is sufficient to diagnose the disease (1, 11), while others emphasize on the evidence of cytologic changes (6, 12). In a study by Gooskens et al. on immunocompromised patients (13), quantitative determination of viral load in bronchial lavage was preferred without considering pathological changes as a measure of lower respiratory infection.

In 50 % of patients with herpetic pneumonia, simultaneous presence of other pathogens can also be noted. Therefore, it appears that herpes simplex virus provides a suitable condition for other infections (14). However, no other pathogen was identified in the current case.

In conclusion, crazy-paving pattern is one of the CT scan findings in herpetic pneumonia. As this pattern is non-specific in immunocompromised patients presenting with symptoms of infectious diseases, early collection of samples from the lung through invasive methods, is strongly recommended. This could help in initiating the anti-viral treatment early and can hence be life-saving.
